# Template Matching and Matrix Profile for Signal Quality Assessment of Carotid and Femoral Laser Doppler Vibrometer Signals

**DOI:** 10.3389/fphys.2021.775052

**Published:** 2022-01-11

**Authors:** Silvia Seoni, Simeon Beeckman, Yanlu Li, Soren Aasmul, Umberto Morbiducci, Roel Baets, Pierre Boutouyrie, Filippo Molinari, Nilesh Madhu, Patrick Segers

**Affiliations:** ^1^PoliToBIOMed Lab, Biolab, Politecnico di Torino, Turin, Italy; ^2^IBiTech-bioMMeda, Ghent University, Ghent, Belgium; ^3^IDLab-imec, Ghent University, Ghent, Belgium; ^4^Photonics Research Group, Center for Nano- and Biophotonics, Tech Lane Ghent Science Park/Campus A, Ghent University-imec, Ghent, Belgium; ^5^Medtronic Bakken Research Center, Maastricht, Netherlands; ^6^Department of Mechanical and Aerospace Engineering, Polytechnic University of Turin, Turin, Italy; ^7^INSERM U970, Université de Paris, Assistance Publique Hôpitaux de Paris, Paris, France

**Keywords:** laser doppler vibrometry (LDV), matrix profile, template matching, logistic regression, signal quality

## Abstract

**Background:** Laser-Doppler Vibrometry (LDV) is a laser-based technique that allows measuring the motion of moving targets with high spatial and temporal resolution. To demonstrate its use for the measurement of carotid-femoral pulse wave velocity, a prototype system was employed in a clinical feasibility study. Data were acquired for analysis without prior quality control. Real-time application, however, will require a real-time assessment of signal quality. In this study, we (1) use template matching and matrix profile for assessing the quality of these previously acquired signals; (2) analyze the nature and achievable quality of acquired signals at the carotid and femoral measuring site; (3) explore models for automated classification of signal quality.

**Methods:** Laser-Doppler Vibrometry data were acquired in 100 subjects (50M/50F) and consisted of 4–5 sequences of 20-s recordings of skin displacement, differentiated two times to yield acceleration. Each recording consisted of data from 12 laser beams, yielding 410 carotid-femoral and 407 carotid-carotid recordings. Data quality was visually assessed on a 1–5 scale, and a subset of best quality data was used to construct an acceleration template for both measuring sites. The time-varying cross-correlation of the acceleration signals with the template was computed. A quality metric constructed on several features of this template matching was derived. Next, the matrix-profile technique was applied to identify recurring features in the measured time series and derived a similar quality metric. The statistical distribution of the metrics, and their correlates with basic clinical data were assessed. Finally, logistic-regression-based classifiers were developed and their ability to automatically classify LDV-signal quality was assessed.

**Results:** Automated quality metrics correlated well with visual scores. Signal quality was negatively correlated with BMI for femoral recordings but not for carotid recordings. Logistic regression models based on both methods yielded an accuracy of minimally 80% for our carotid and femoral recording data, reaching 87% for the femoral data.

**Conclusion:** Both template matching and matrix profile were found suitable methods for automated grading of LDV signal quality and were able to generate a quality metric that was on par with the signal quality assessment of the expert. The classifiers, developed with both quality metrics, showed their potential for future real-time implementation.

## 1. Introduction

The aorta and large central arteries fulfill key physiological functions in the circulation, whereby their structure is apt to their function. They consist of complex composite soft tissues, concentrically organized in lamellar units, where sheets of elastin intertwine with layers of vascular smooth-muscle cells in a matrix of collagen and other proteins composing the extra-cellular matrix (Wolinsky and Glagov, 1967). This allows the aorta and large arteries to distend when the heart contracts and blood is ejected into the aorta and store elastic energy in the arterial wall, which is used during the relaxation phase of the heart to maintain blood pressure and drive the perfusion of organs and tissues. This function is also referred to as the windkessel or buffering function of the large arteries, and ensures that the pulsatile blood flow generated by the heart is transformed into a near steady flow when reaching the smaller arteries (Westerhof et al., 2009). It prevents excessive maximal (systolic) and too low minimal (diastolic) blood pressure. Arterial stiffening leads to a loss of this buffering function with detrimental effects on nearly all organ systems, and especially low resistance organs such as the brain, the kidneys, and the heart itself (Chirinos et al., 2019). Arterial stiffening has received large attention over the past three decades, and there is a consensus that assessment of arterial stiffness is especially relevant in the assessment of an individual's risk for cardiovascular disease and death (Laurent et al., 2006; Vlachopoulos et al., 2010).

Because of the distensible nature of arteries, cardiac contraction generates a wave (detectable as a change in pressure, flow, or arterial diameter). This wave initially propagates from the heart to the periphery, but increases in complexity as it interacts on its way with the branching arterial tree and gets shaped because of wave reflection and transmission (O'Rourke and Kelly, 1993; Mitchell et al., 2004, 2011; Chirinos et al., 2019). The wave speed, or pulse wave velocity (PWV), is directly linked with the distensibility of the arteries (the stiffer the artery, the higher PWV) (Bramwell and Hill, 1922), and the current clinical standard method to measure arterial stiffness is by measuring the pulse wave velocity (Segers et al., 2020). In essence, the method is simple and straightforward: one detects the pulse at two locations a distance *dx* apart, and from the time delay, *dt*, between the signals, one gets PWV = *dx*/*dt*. Despite the simplicity of the concept, there are still many hurdles in measuring PWV in practice, mainly related to the non-availability of sites to directly measure the pulse along the path of the aorta in a non-invasive way and without the need for clinical scanners (Segers et al., 2020). Accessible sites closest to the aorta are the neck (carotid artery) and groin (femoral artery) and carotid-femoral PWV is considered the best possible proxy for aortic PWV (Laurent et al., 2006).

Several sensors can be used to detect the pulse in the neck and groin (Pereira et al., 2015; Segers et al., 2020), including applanation tonometry, ultrasound (pulsed Doppler recordings), or accelerometers. Motivated by the relatively high cost of equipment, the required level of expertise by the operator, or the contact-based nature of the measurement, we and others have explored the use of Laser-Doppler Vibrometry (LDV) to detect the motion of the skin atop the carotid and/or femoral arteries in response to the passage of the arterial pulse (Morbiducci et al., 2007b; De Melis et al., 2008; Scalise and Morbiducci, 2008; Campo and Dirckx, 2011; Kaplan et al., 2012). To eliminate motion drift and amplify the fast displacements associated with the arrival of the foot of the pulse (Morbiducci et al., 2007b), we have been using skin acceleration as the basic signal from which to derive time delays between the neck and groin for measuring carotid-femoral PWV.

The feasibility of the method has been shown using industrial-type LDV sensors (De Melis et al., 2008), and we have been working on the design and development of a multi-beam handheld device. The core of the device is a silicon photonics chip integrated into a micro-optical system which allows for flexible and compact multi-array designs (Li et al., 2013, 2020). A first prototype (consisting of 2 connected yet separable handheld pieces to measure in the neck and groin with each 6 laser beams) was developed within the context of the H2020-funded project CARDIS and included a clinical feasibility study whereby carotid-femoral PWV was assessed in 100 patients and compared with a reference method based on applanation tonometry (Marais et al., 2019). Measurements were performed with a minimal visual feedback during the measurements and all the analyses were carried out in an off-line modality.

A next generation version of the device is under development and will provide real-time measurement of carotid-femoral PWV. To do so, we need a real-time assessment of the quality of incoming data to decide whether or not data records are of an acceptable quality for subsequent processing. This is, however, not a trivial assessment as there is little reference as to what makes LDV signal recordings appropriate for PWV estimation.

The aim of this study is, therefore, to identify a strategy to objectively and automatically assess the LDV-signal quality and set criteria for future use of this technology in arterial pulse detection. To do that we will use the existing CARDIS database of LDV recordings at the carotid and femoral measurement sites and subject them to two different strategies: the template matching and the matrix profile will be tested for (1) analyzing the nature and achievable quality of the recorded signals, and (2) exploring models for automated classification of LDV-signal quality.

## 2. Materials and Methods

### 2.1. The CARDIS Device

**Figure 1:**
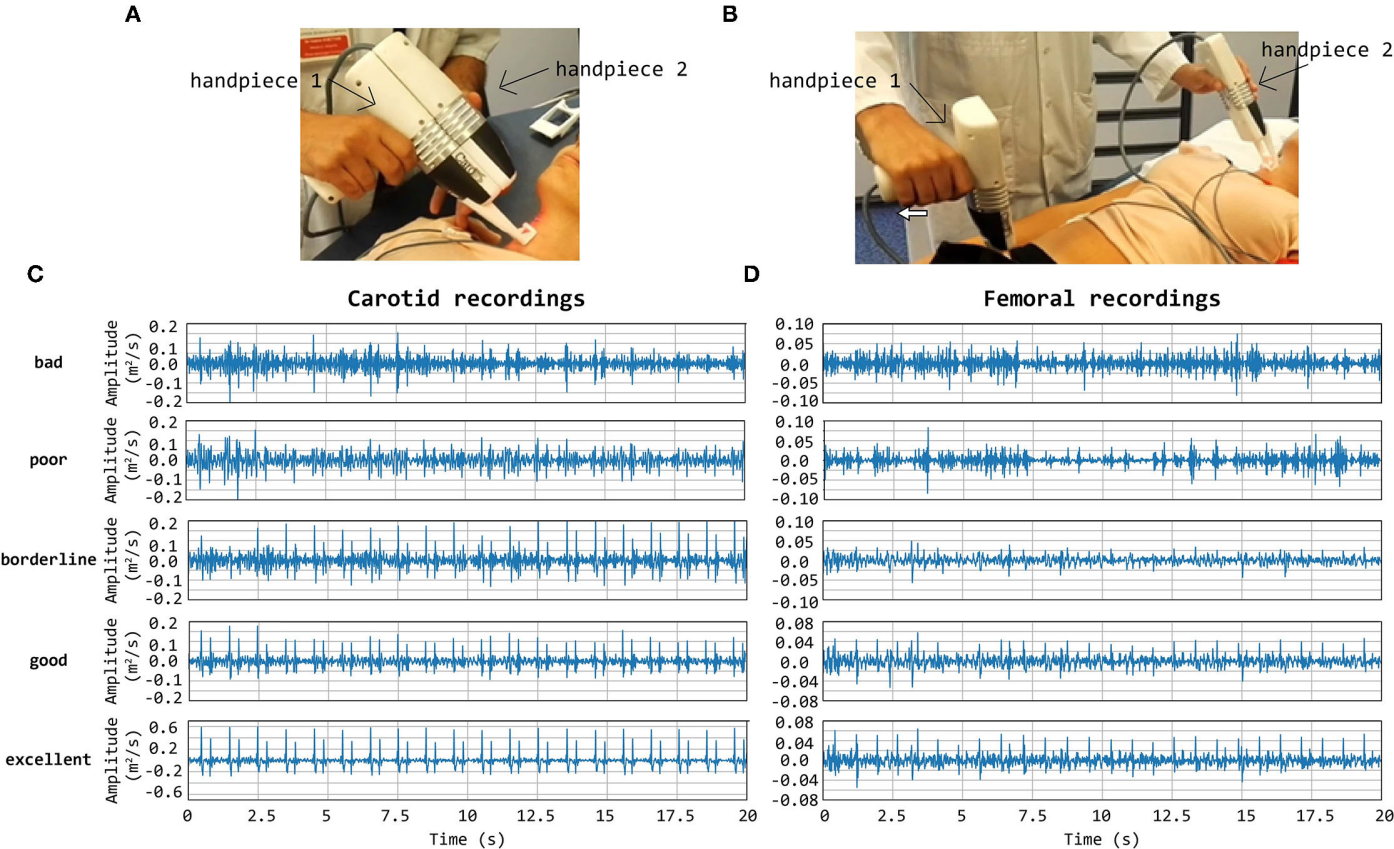
CARDIS device in configuration to measure carotid-femoral **(A)** and local carotid PWV **(B)**. **(C,D)** display representative tracings on the carotid **(C)** and femoral **(D)** measuring site receiving a visual grading score of 1–5. Especially in the excellent tracings, the foot-of-the-wave waveforms are clearly visible, with the same for the dicrotic notch waveforms in the local carotid case.

Technical details on the optics and overall design of the CARDIS device have been described in Li et al. (2020). Briefly, the device consists of two handpieces (handpiece 1 contains the handgrip of the device, handpiece 2 is the add-on part of the device: we refer to Figure 1 for an illustration of the device and the positioning of the handpieces), each sending out 6 laser beams (wavelength 1,550 nm), positioned along a line and 5 mm apart. The handpieces can be used separately for measurement of carotid-femoral PWV or attached to measure signals on locations 25–50 mm apart, e.g., to locally measure pulse wave propagation along the carotid artery. A retro-reflective tape is attached to the skin at the measurement location to enhance the reflection of the laser light, and the device is equipped with a spacer to ensure an appropriate optical focus distance and to stabilize measurements.

### 2.2. Study Population and Available Database

The data used in this study were acquired with a clinical feasibility study in 100 patients, conducted at the Hpital Europen Georges Pompidou (HEGP) in Paris, France, to assess the ability of the CARDIS device to measure signals in a configuration with simultaneous carotid-femoral or carotid-carotid recordings. Patients were in the age range 19–85 and presented with mild to stage 3 hypertension, controlled or not (Marais et al., 2019). For each subject, 4–5 datasets, each consisting of 20 s traces on 12 channels measured with the two handpieces, were acquired. In detail, the analyzed database was made of 410 datasets (4,920 waveforms) from carotid-femoral recordings, and of 407 datasets (4,884 waveforms) from carotid-carotid recordings. Raw IQ (In-phase and quadrature) LDV-data were acquired at a sampling frequency of 100 kHz, and LDV-displacement data were downsampled to 10 kHz upon demodulation. A low-pass filter with a cut-off frequency of 30 Hz was applied to LDV displacement data, which were differentiated two times to yield acceleration. The same low-pass filtering strategy was applied after each differentiation operation.

### 2.3. Visual Scoring of the Data

A graphical interface displaying all the LDV acceleration signals derived from the six channel recordings per handpiece was implemented in the MATLAB environment (The MathWorks, Naticks MA, US). The acceleration signals were visually scored by an expert operator (Segers P.) on a 5-level grade scale taking values *Q*_vis_ according to Table 1.

**Table 1:** The 5-levels grade scale taking values *Q*_vis_.

**Quality score *Q*_vis_**	**Quality**	**Description**
Score 1	Bad	Acquisition with no evidence of repeatable features that may be linked to the detection of a pulse
Score 2	Poor	Very noisy acquisition not suitable for analysis, but with identifiable pulses within the noisy trace
Score 3	Bordeline	Acquisition affected by noise but presenting clear repeatable patterns. Advanced signal processing algorithms could remove the noise and allow to detect the foot of the pulse wave with reasonable affordability
Score 4	Good	Acquisition with sharp and pronounced peaks at the foot (and dicrotic notch), with relatively low noise levels between successive pulse peaks
Score 5	Excellent	Acquisition with very sharp and pronounced peaks at the foot (and dicrotic notch), with low noise levels in between the peaks. Signals of textbook quality

Note that the presence of brief artifacts in the 20 s acquired traces was not used as a criterion to score the signal quality. As such, signals qualified as excellent may still demonstrate a brief episode of poor data. Overall, the femoral data were of a markedly lower *Q*_vis_ “quality” than traces recorded at the carotid artery, which impacted the rating. Therefore, the *Q*_vis_ quality score of 3 (borderline) was given to femoral traces that appeared to be of a much lesser quality than *Q*_vis_ = 3 rated carotid traces. Such a borderline score was assigned when 5–10 beats were discernible in the signal. Representative carotid and femoral signals receiving the different scores are displayed in Figure 1.

### 2.4. Template Matching

**Figure 2:**
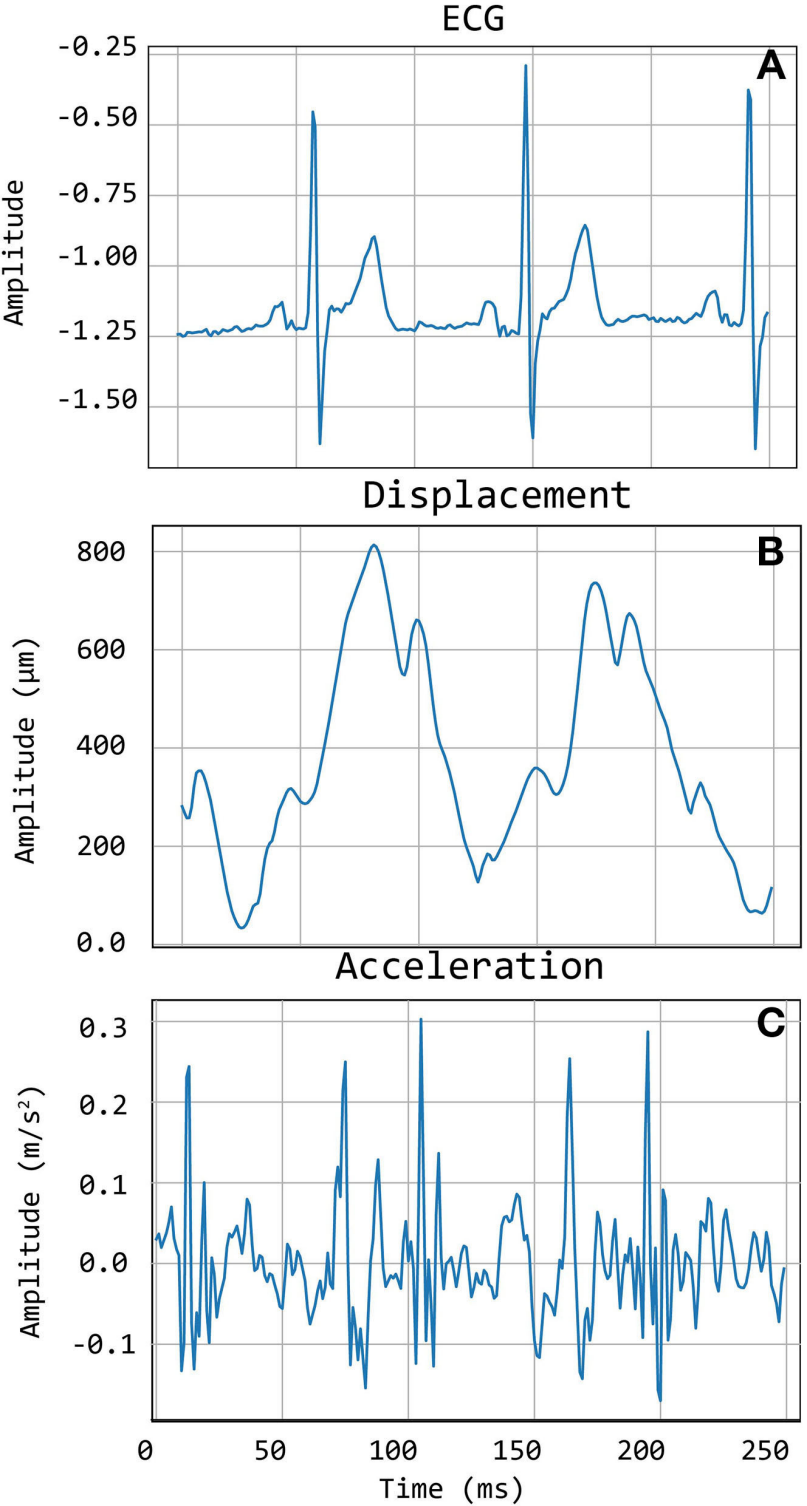
An example of Cardis data. **(A)** shows the ECG signals, **(B)** shows the displacement signal, and **(C)** the corresponding acceleration signal.

Template matching technique is an effective approach for the automatic detection of *a priori* identified patterns in signal recordings (Jiun-Hung et al., 2003; Won-Du and Chang-Hwan, 2014) and images (Omachi and Omachi, 2007). A good-quality carotid LDV acceleration signal presents two sharp peaks for each heartbeat: the first peak corresponds to the systolic rapid upstroke of pressure and demarcates the foot of the arterial pulse; the second peak denotes the wave that is generated at the moment of closure of the aortic valve (the dicrotic notch). The LDV-femoral recording is devoid of clearly identifiable features related to the dicrotic notch because of the distance of the measurement site from the heart, whose final effect is filtering the recorded LDV pulses, in the femoral artery. An example of displacement, acceleration, and ECG signals together are shown in Figure 2.

#### 2.4.1. Constructing the Templates

High-quality carotid and femoral LDV-acceleration traces were adopted for template construction. Traces with visual score values *Q*_vis_ of 4 and 5 were selected. To avoid subject-specific biasing in template construction, only one 20 s recording (from the acquired channel with the highest *Q*_vis_) per subject was selected. Based on these selection criteria, 135 carotid LDV-acceleration traces from 20 different subjects and 40 femoral LDV-acceleration traces from 10 different subjects were identified as suitable for template construction in the CARDIS dataset. The selected carotid LDV-acceleration traces were from both handpieces.

**Figure 3:**
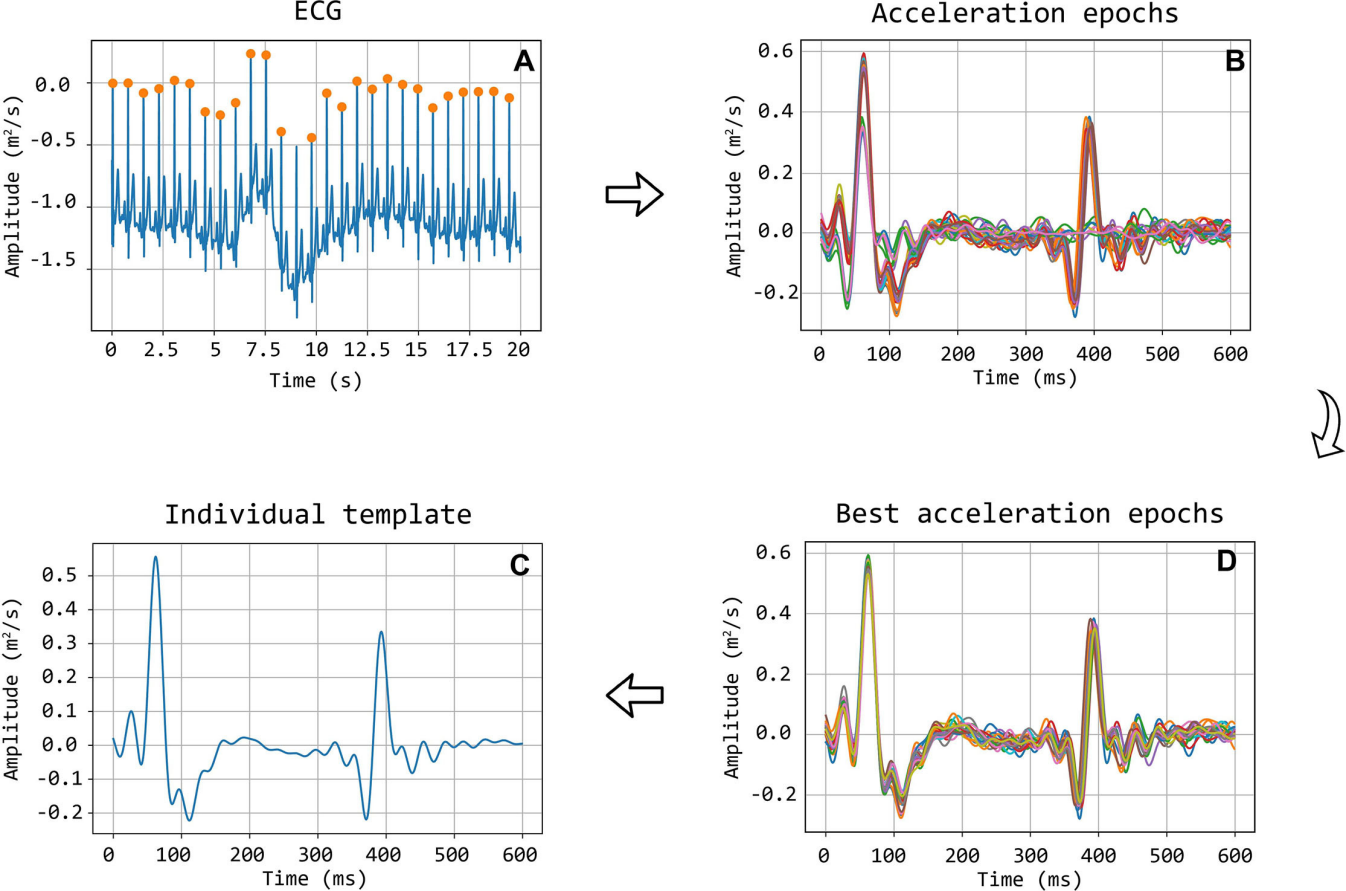
Workflow detailing the construction of the template. Illustration of selection of good quality epochs using the correlation coefficient. **(A)** ECG signal with detected R peaks, which are used to segment the acceleration signal into heartbeat epochs **(B)**. After the correlation matrix analysis, only the good epochs are maintained **(D)**. In **(C)** the final individual template, calculated as the average of the good epochs, is displayed.

**Figure 4:**
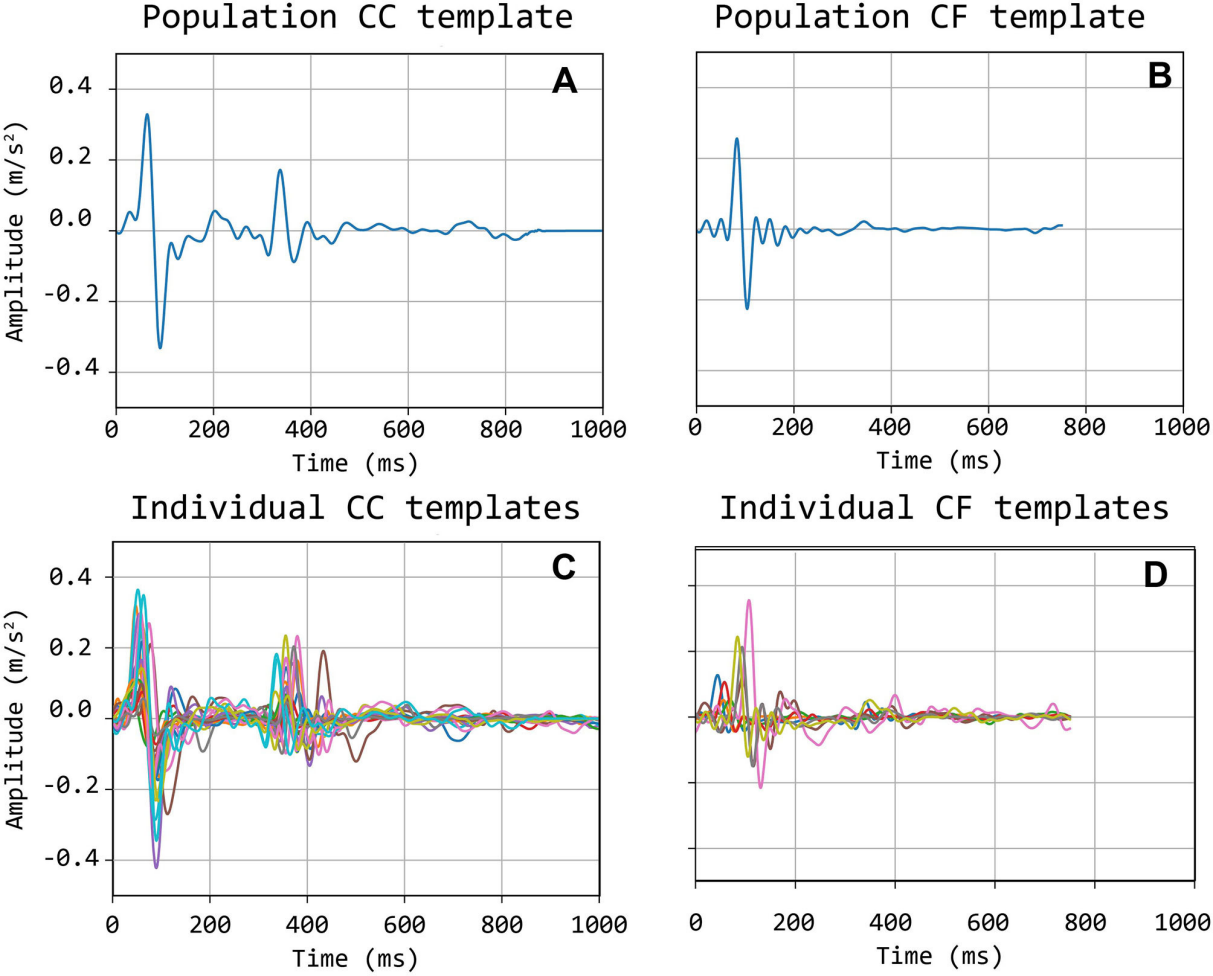
Bottom **(C,D)**: individual carotid (CC) and femoral (CF) templates; Top **(A,B)**: population-average carotid and femoral templates.

The selected traces, characterized by the presence of sharp and pronounced peaks at the foot (and dicrotic notch for carotid recordings), were then segmented in epochs, each one corresponding to a single heartbeat. LDV-acceleration trace segmentation was carried out using ECG synchronous recordings (available for each subject in the CARDIS dataset, on which automatic R-peak detection was carried out, refer to Figure 3). Over each LDV trace, single epochs were then defined within a time interval within the occurrence of two consecutive R peaks in the ECG trace (Figure 3A). By construction of the visual inspection classification, some of the identified single epochs might still not be of adequate quality for template construction, because of the presence of short-time artifacts/noise (Figure 3B). The lower quality single epochs in an LDV-acceleration trace were identified according to the following strategy: (1) for each LDV segmented trace, a correlation matrix *R*_*ij*_ was built up, each element of the matrix being the Pearson-correlation coefficient between epochs i and j, used as a measure of their shape similarity; (2) a threshold value of the correlation coefficient was defined and single epochs with an average correlation coefficient with all the other epochs lower than the threshold was discarded, since they were not sufficiently similar in shape to the other epochs in the recorded trace (Figure 3C); (3) for each LDV-acceleration trace, an “individual template” was built up by averaging only the identified highly correlated epochs (Figure 3D); (4) by adopting the same approach with the carotid and femoral LDV-acceleration traces, the final carotid and femoral “population templates” were obtained (Figure 4).

**Figure 5:**
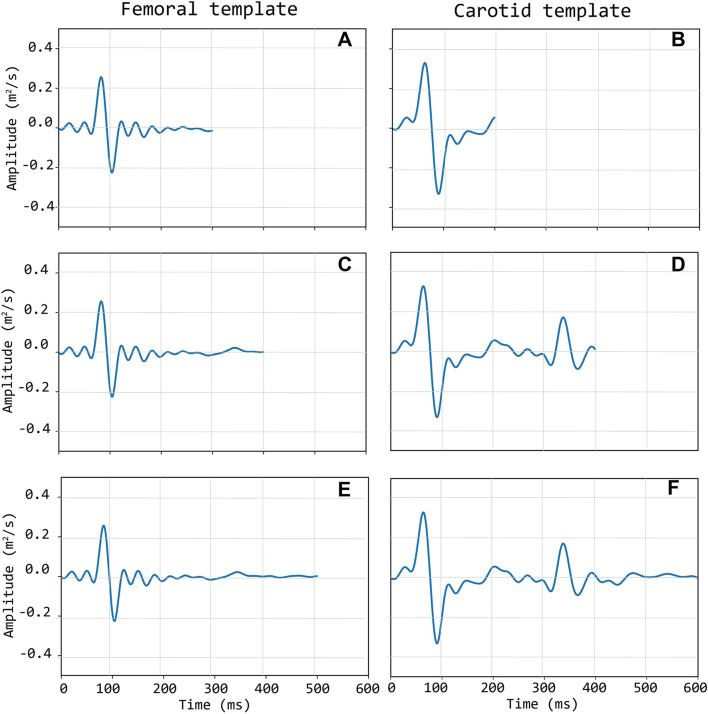
Left column **(A,C,E)**: three different length of the femoral template; Right column **(B,D,F)**: three different lengths of the carotid template.

Template construction is based upon the definition of a strategy to treat the issue of the different time length of single epochs (intra-individual RR variability) (Jensen-Urstad et al., 1997; Zhang, 2007) and the individual templates as well. Hence, the time length of single epochs should be defined on the basis of what the template should represent. In the case under study, the carotid LDV-acceleration template longer than 350 ms will include by construction the foot of the wave (first peak) and the dicrotic notch (second peak). In this study, we speculate that a carotid LDV template incorporating the second peak may degrade in performance, as the distance between the two peaks is (intra-individually and inter-individually) variable. In Figure 5, carotid and femoral LDV-acceleration templates constructed for different (predefined) time length are displayed. In detail, time lengths of 300, 400, and 500 ms were considered for the femoral LDV-acceleration template, and time lengths of 200, 400, and 600 ms for the carotid LDV-acceleration template. The impact of the time length in the LDV template performance when used for the automatic assessment of the quality of the CARDIS data was evaluated.

#### 2.4.2. Template Matching and Beat Selection

**Figure 6:**
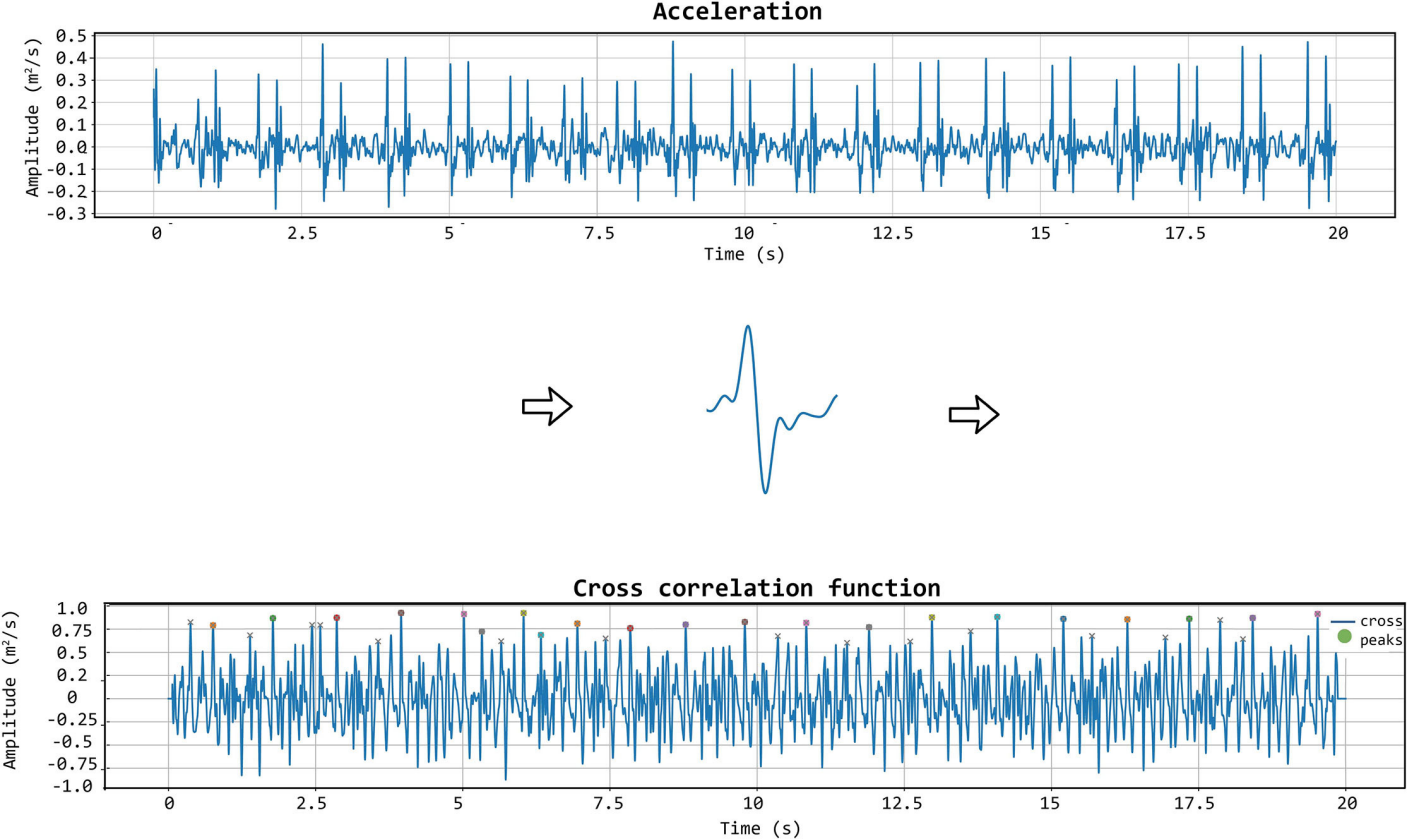
The template matching algorithm is shown. The chosen template (displayed in the middle row of the figure) is iteratively correlated with the acceleration signal to get the cross-correlation function. In that function, the appropriate peaks are then identified.

The matching between the templates and the LDV-acceleration traces in the CARDIS dataset was performed by applying a local moving-window function calculating the Pearson's correlation coefficient between the LDV template and the 20 s-long acceleration trace at each time step, as displayed in Figure 6. The locations of peaks in the time series resulting from this moving-window cross-correlation operation identify the time instants where the sliding template is similar to a segment of the LDV-acceleration trace.

Setting a threshold for the value of the cross-correlation coefficient then demarcates the correspondence level above which segments of the LDV-acceleration trace can be considered similar to the template. Based on the set threshold value, single segments corresponding to single heartbeats in the LDV trace can be considered of sufficient or not sufficient quality.

**Figure 7:**
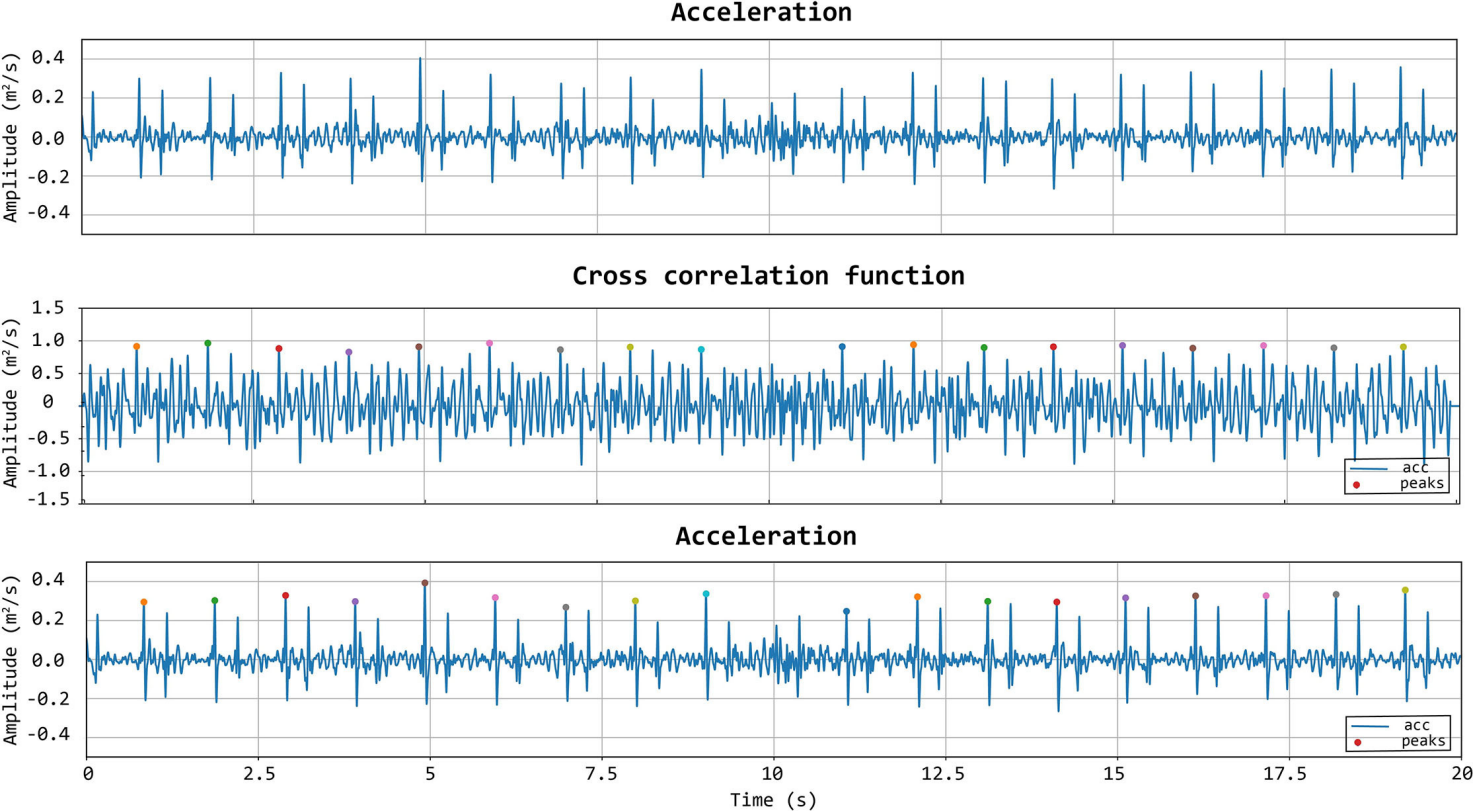
First row: acceleration signal. Second row: normalized cross-correlation function and its maximum values of the acceleration signal with the femoral template of 500 ms. Last row: acceleration signal with the detected peaks using the template matching method.

To further improve the identification of high-quality heartbeats in the LDV recorded traces, two further selective criteria were added. First, all the LDV-acceleration peaks in the recorded trace with an amplitude lower than 80% of the average peak amplitude were not considered. Then, if two successive peaks were detected within a time window shorter than 500 ms, the second peak was discarded and only the first one was considered. The latter criterion was adopted to avoid the dicrotic notch detection (second peak), especially when the shorter carotid template was used. An explanatory example of peak detection, presenting the LDV-acceleration trace, the moving-window cross-correlation function, and detected peaks are displayed in Figure 7.

#### 2.4.3. LDV Traces Classification Based on Template Matching-Finding Threshold Values

The performance of the template matching algorithm in classifying the quality of the CARDIS dataset was evaluated by comparison with visual score classification, according to the following scheme: acceptable heartbeat (label 1), corresponding to *Q*_vis_ values 4 or 5; not acceptable heartbeat (label 0) corresponding to *Q*_vis_ values 1 or 2. Signals with *Q*_vis_-values of 3 are discarded in this analysis as these signals are difficult to assign an absolute and correct classification (refer to discussion). The template matching-based classification, as also mentioned before, depends upon the threshold value for the moving-window correlation function and the number of detected heartbeats in the LDV-acceleration trace, which have to be appropriately set.

In this study, we considered: true positive (TP) an acceptable LDV trace (based on *Q*_vis_ classified by the template matching as acceptable; false negative (FN) an acceptable LDV trace classified by the template matching as not acceptable; true negative (TN) an unacceptable LDV trace classified by the template matching as not acceptable; false positive (FP) an unacceptable LDV trace classified by the template matching as acceptable. On this basis, sensitivity and specificity values of the classifier are defined as:


(1) $$\begin{array}{l} \text{Sensitivity}=\frac{TP}{TP+FN} \end{array}$$

and


(2) $$\begin{array}{l} \text{Specificity}=\frac{TN}{TN+FP} \end{array}$$

Sensitivity and Specificity were then used to build up the Receiver Operating Characteristic (ROC) curves and their area under the curve (AUC) was used to assess the performance of the classifier. Moving-window cross-correlation coefficient threshold values and the number of detected heartbeats yielding the highest AUC were defined on the complete CARDIS dataset and this for each one of the template lengths in time.

#### 2.4.4. LDV Traces Classification Based on Template Matching-Defining Quality Score and Testing on the CARDIS Dataset

Once the best performing carotid and femoral templates time length and the associated moving-window cross-correlation threshold values were identified, a quality score (*Q*_TM_)was estimated for each 20 s LDV-trace recording, based on two main features.

The first feature (*Q*_1_) is the number of the detected acceleration peaks (*n*_peaks_), normalized with the maximum expected number of peaks or heartbeats in the 20 s LDV-trace recording (max_peaks_). This value was empirically set equal to 26 to ensure a maximal feature value of 1 in the investigated database:


(3) $$\begin{array}{l} Q_{1}=\frac{n_{\text{peaks}}}{{\text{max}}_{\text{peaks}}} \end{array}$$

The second feature (*Q*_2_) is defined as the average time delay between the occurrence of a maximum value of each LDV-acceleration epoch in the recorded trace and the occurrence of the peak value on the template (*d*_*peak*_*n*__), normalized to the template time length (*N*):


(4) $$\begin{array}{l} Q_{2}=\frac{\sum_{n=1}^{n_{\text{peaks}}}\left(1-\frac{d{\text{peak}}_{n}}{N}\right)}{{\text{max}}_{\text{peaks}}} \end{array}$$

When the peaks in the template and in each LDV epoch are all perfectly aligned, and when all the peaks in the LDV-trace are detected (i.e., *Q*_1_ = 1), feature *Q*_2_ is equal to 1, indicating good quality of the LDV trace recording. The final score based on template matching can be computed as the mean value of the partial scores *Q*_1_ and *Q*_2_:


(5) $$\begin{array}{l} Q_{\text{TM}}=\frac{1}{2}\left(Q_{1}+Q_{2}\right) \end{array}$$

By construction, the score *Q*_TM_ was set up so that the value is within the range [0, 1] (with *Q*_TM_ = 0 representing the worst possible signal quality and *Q*_TM_ = 1 indicating that the signal is of excellent quality). *Q*_TM_ was calculated for all the traces in the CARDIS database and compared to the corresponding assigned visual score *Q*_vis_, which is treated as the ground truth.

#### 2.4.5. A Logistic Regression Model for Signal Classification Based on Template Matching

*Q*_TM_ Was a heuristically derived quality metric with equal weighting on the sub-components. Now we use logistic regression models to find a better weighting of the contributions of *Q*_1_ and *Q*_2_, and automatically map this to a predicted quality of the signal. Logistic regression models are chosen since they can be well applied to binary classification problems and are typically used in medical research (Domínguez-Almendros et al., 2011; Austin and Steyerberg, 2012; Nick and Campbell, 2012) when a two-class classifier is required. These predictions were then compared to the ground truth labels (given by the visual scores).

Logistic regression models were trained and tested with the two template-matching derived scores (Equations 3 and 4) as features, on both carotid and femoral LDV-acceleration traces. For this purpose, again the LDV traces visually scored with *Q*_vis_ equal to 1 or 2 were labeled 0, and LDV traces visually scored with *Q*_vis_ equal to 4 or 5, were labeled 1. Again, signals with a Qvis score of 3 were not included in the analysis.

The data available in the CARDIS database was split such that 80% was used for training the logistic regression model and the remaining 20% used for testing purposes. The training-testing set partition was randomly iterated 1,000 times while storing the model accuracy every iteration, so that the overall accuracy distribution of the logistic regression model approach could be assessed.

Of note, all features used to train logistic regression models were normalized *via* standardization. This allowed the logistic regression-model coefficients to be interpreted as the corresponding feature weights, granting information about which feature was most influential in labeling an LDV trace.

The accuracy distributions of logistic regression models trained on template-matching and the later discussed matrix-profile derived features were evaluated.

### 2.5. Matrix Profile

The matrix profile is a data structure that annotates a time series (Yeh et al., 2018; Zhu et al., 2020). It allows for exact, simple, and fast (Zhu et al., 2017b) similarity search or discord discovery and is among the state-of-the-art techniques in the field of discrete time-series analysis (Zhu et al., 2017a; Madrid et al., 2019). The matrix profile has been used in processing biological signals like EEG (Mueen et al., 2009), ECG, and gait cycles (Zhu et al., 2020). It was applied in this study to accurately identify recurring waveforms in the LDV-acceleration data. Every such waveform is a subsequence of the original sequence or time series. These subsequences, taken together, are collectively called a motif. We gauged the quality of an LDV-measurement *via* several features determined by its best motif. The strength of the matrix profile lies in the fact that it does not require a template or other input parameters except for the length *m* of the desired motif subsequences. Analogous to the template matching analysis, waveforms were subsampled to 1 kHz. We set *m* to 200 ms, similar to the optimal length of the template described in previous sections.

#### 2.5.1. Signal Classification Based on the Matrix Profile

A quality metric (*Q*_MP_) was constructed based on three features of the matrix profile-generated motif as seen in Equation (6). This metric was constructed so that its possible values lie between 0 and 1.


(6) $$Q_{\text{MP}}=A_{\text{MP}}\;t_{\text{d,MP}}\;n_{\text{MP}}$$

The first feature used in calculating (*Q*_MP_) is the average relative maximum amplitude of a subsequence in the motif (*A*_MP_) computed as in Equation (7). The maximum amplitude of subsequence *A* was compared with the maximum amplitude of the reference subsequence *A*_ref_. This reference is the first subsequence identified by the matrix profile (the minimum of the matrix profile) and subsequently included in the motif. In good quality measurements, most maximum amplitudes of subsequences in the motif were similar.


(7) $$\begin{array}{l} A_{\text{MP}}=\frac{1}{n_{\text{mtf}}}\overset{n_{\text{mtf}}}{\underset{n=1}{\sum }}\frac{A}{A_{\text{ref}}} \end{array}$$

The second feature, the average relative time-instant of the subsequence peaks in the motif (*t*_d,MP_), is computed as in Equation (8). The time-instant of the subsequence peak was compared with that of the reference. This value was then normalized over the length of the subsequence *m*. Ideally, all subsequences in the motif represent the same heartbeat-related waveform with peaks at similar time instants. For poor quality signals, these time instants tended to randomly vary over the length of the subsequence.


(8) $$\begin{array}{l} t_{\text{d,MP}}=\frac{1}{n_{\text{mtf}}}\overset{n_{\text{mtf}}}{\underset{n=1}{\sum }}\left(1-\frac{d_{\text{peak}}}{m}\right) \end{array}$$

Finally, the third feature (*n*_MP_) was calculated as the expected amount *n*_exp_ vs. the effective amount *n*_mtf_ of subsequences in the motif, shown in Equation (9). *n*_exp_ was estimated based on a discrete-Fourier-transform analysis of the entire signal recording. More specifically, the peak corresponding to the heartbeat during the measurement was identified as the most prominent peak in the signal spectrum, in the range 0.5–1.5 Hz. The effective amount of subsequences in the motif *n*_mtf_ was based on how many heartbeats the matrix-profile technique was able to pick up.


(9) $$\begin{array}{l} n_{\text{MP}}=\frac{n_{\text{mtf}}}{n_{\text{exp}}} \end{array}$$

Before a subsequence is included in the motif, three criteria decide the inclusion: (1) If a subsequence maximum amplitude was lower than 0.8 times the reference maximum amplitude it was excluded from the motif. (2) If the time instant of the peak deviated 30 ms or more from that of the reference, the subsequence was also removed from the motif. (3) If two subsequences were closer than 0.8 times the expected time delay between two subsequent heartbeats, the one with the lower matrix-profile value (higher similarity to the reference) of the two was preserved, the other was removed. The applied thresholds levels were determined empirically from excellent and poor quality signals.

**Figure 8:**
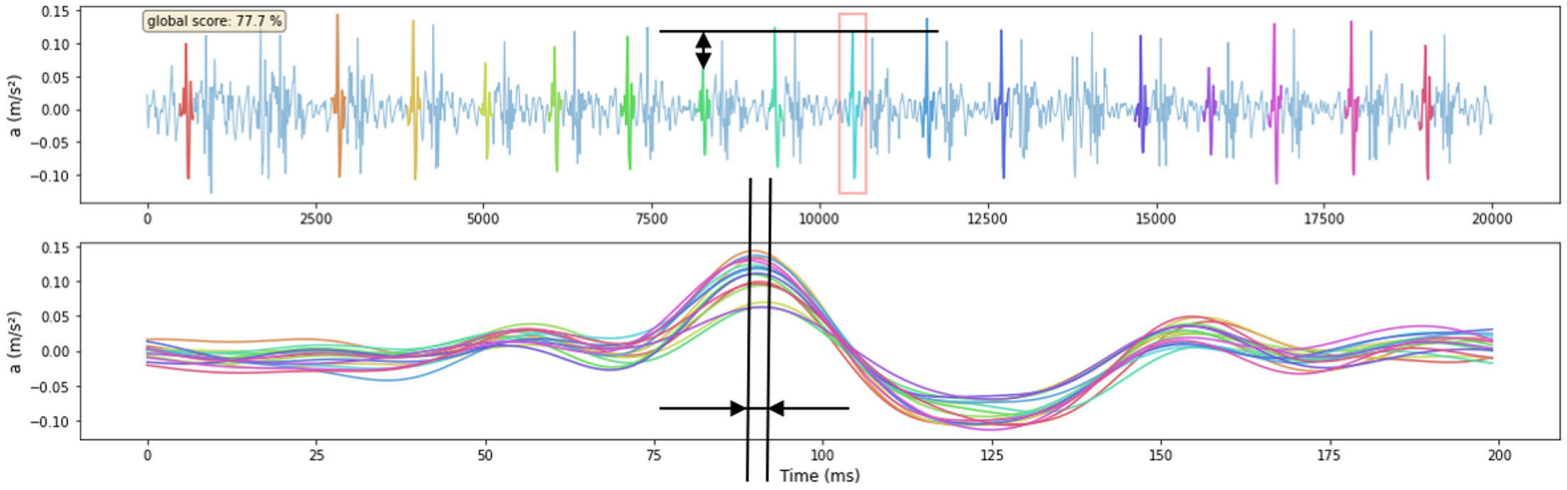
Example of one signal being scored by the features that are derived from the Matrix-Profile-identified motif. The amplitude feature of one subsequence is shown in the upper figure, the reference is indicated with a red square. The time-instant of subsequence peak feature is shown in the lower figure where all subsequences shown in the upper figure are time-aligned. The signal score is shown in the upper left corner of the upper figure. The visual score of this signal is 4.

Figure 8 shows an example of a signal being scored by first finding the motif so that as many heartbeats as possible are present within it, then calculating the features of that motif. Both the relative amplitude and time-instant of subsequence peak features of one subsequence in the motif are indicated in the figure.

The auto-generated matrix-profile-based quality metric was computed for all carotid-carotid and femoral-carotid datasets and results were compared to the visual scores.

#### 2.5.2. A Logistic Regression Model Based on the Matrix Profile

Similar to template matching, we also designed logistic regression models using the previously discussed matrix-profile derived features. These models allow for more freedom in weighting the features to come to a better classification result. Models were trained and tested on the three features mentioned above. Signals were labeled and available data was split into training and testing sets analogous as in the previously discussed template-matching case.

### 2.6. Relation Between Signal Quality and Physiological Variables

Finally, we investigated the existence of possible associations of quality of the LDV-acceleration traces with age, body mass index (BMI), and systolic blood pressure. The statistical analysis was performed using *Q*_MP_ as a quality score variable. In detail, the existence of a linear correlation was tested using the Pearson-correlation coefficient on both CC and CF datasets, with data analyzed per handpiece. For all analyzes, significance was assumed for *p* < 0.05.

## 3. Results

### 3.1. Visual Scoring

#### 3.1.1. Carotid-Carotid Measurements

**Figure 9:**
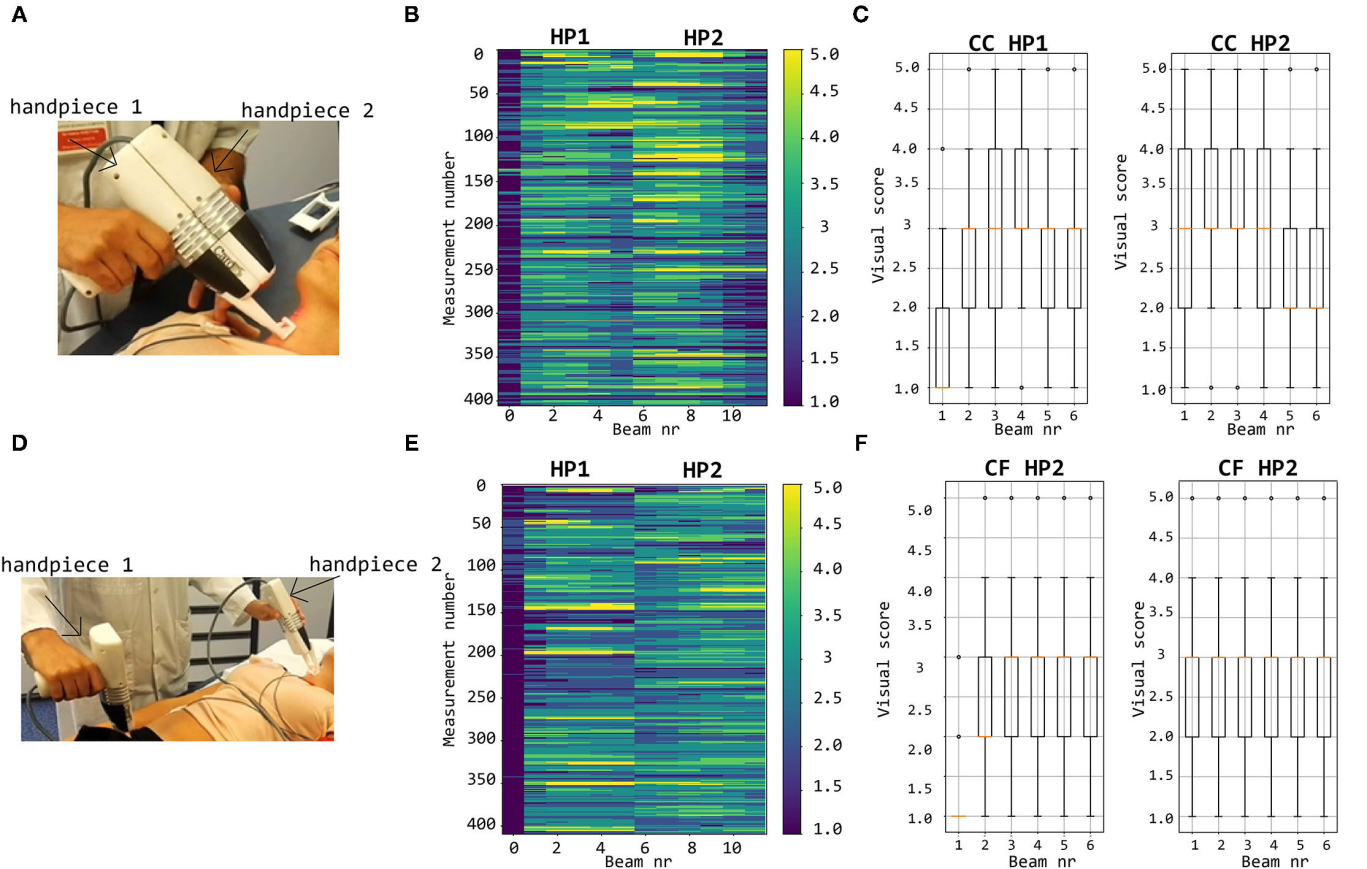
Top row: visual scoring of signals measured with handpieces 1 and 2 during local carotid measurements **(A)** with overall grades shown in **(B)** and box plots and mean values per channel in **(C)**. Bottom row: visual scoring of signals measured during carotid-femoral PWV measurements **(D)** with overall grades shown in **(E)** and box plots and mean values per channel in **(F)**.

By visual inspection, about 12% of all LDV-acceleration traces were qualified as bad and close to 30% as poor (Figure 9B). This implies that about 42% of the recorded LDV traces were evaluated to not be of sufficient quality for further analysis. About 22% of all recordings were scored from good to excellent and are deemed suitable for further analysis. About 37% of the traces were visually scored borderline, i.e., these traces might be of sufficient quality for further analysis with advanced processing. The number of LVD traces scored with *Q*_vis_ 4 or 5 and recorded using handpiece 2 was higher than the number using handpiece 1. For handpiece 1, channel 1 scored almost systematically very low; the best channels were channels 3 and 4. For the second handpiece, the best channels were channels 2 and 3.

#### 3.1.2. Carotid-Femoral Measurements

The bottom row of Figure 9 illustrates that, concerning femoral LDV-acceleration traces (handpiece 1), 20% of all recordings were qualified as bad, and another 32% as poor, meaning that over 50% of all recordings is not usable for analysis. About 15% of the measured signals get a score good to excellent, deemed immediately suitable for analysis. The best channels are channels 3 and 4 with 21.9% (beam 3) and 19.5% (beam 4) of the recordings good to excellent. For handpiece 2 (carotid recordings), about 20% gets a score good to excellent. This is less than what was obtained for handpiece 2 for the carotid-carotid recordings, where close to 25% of all recordings were rated good to excellent. On the other hand, less signals received grades 1 and 2. The best channels are channels 4 (24.9%) and 5 (24.1% of the recordings scoring good to excellent).

### 3.2. Template Matching

#### 3.2.1. Carotid-Carotid Measurements

From the analysis carried out on the complete CC dataset, it emerged that using the carotid template of 200 ms length guarantees the best performance in terms of specificity, setting the cross-correlation threshold to 0.74 and the minimum number of detected heartbeats per trace to 15 (AUC = 0.89, sensitivity 74%, specificity 89%; template of 400 ms length: AUC = 0.89, sensitivity 81%, specificity of 83%; template of 600 ms length: AUC = 0.92, sensitivity 87%, specificity 86%). For each template length, the corresponding confusion matrix is presented in Table 2. The adoption of specificity for the evaluation of the performance of the template matching strategy was dictated by the need of maximizing the removal of LDV traces with inadequate quality. More in detail, it emerged that in general, the template matching performed excellently in correctly classifying visual scores 1 and 5, while accuracy decreased for visual scores 2 and 4 (Table 2). Interestingly, using the shorter template length of 200 ms led to a score of 42% of the LDV acceleration traces visually scored 3 (borderline) as acceptable data.

**Table 2:** Confusion matrices of signal classification done by the hand-engineered classification model constructed with template matching.

	**Template of 200 ms**		**Template of 400 ms**		**Template of 600 ms**	
**Carotid recordings**	**TM score 0(%)**	**TM score 1(%)**	**TM score 0(%)**	**TM score 1(%)**	**TM score 0(%)**	**TM score 1(%)**
Score 1	97	3	97	3	97	3
Score 2	86	14	86	14	82	18
Score 3	58	42	55	45	44	56
Score 4	29	71	30	70	15	85
Score 5	8	92	19	81	6	94

*Signals classified in this table were measured at the carotid and the templates used were the carotid population templates*.

**Figure 10:**
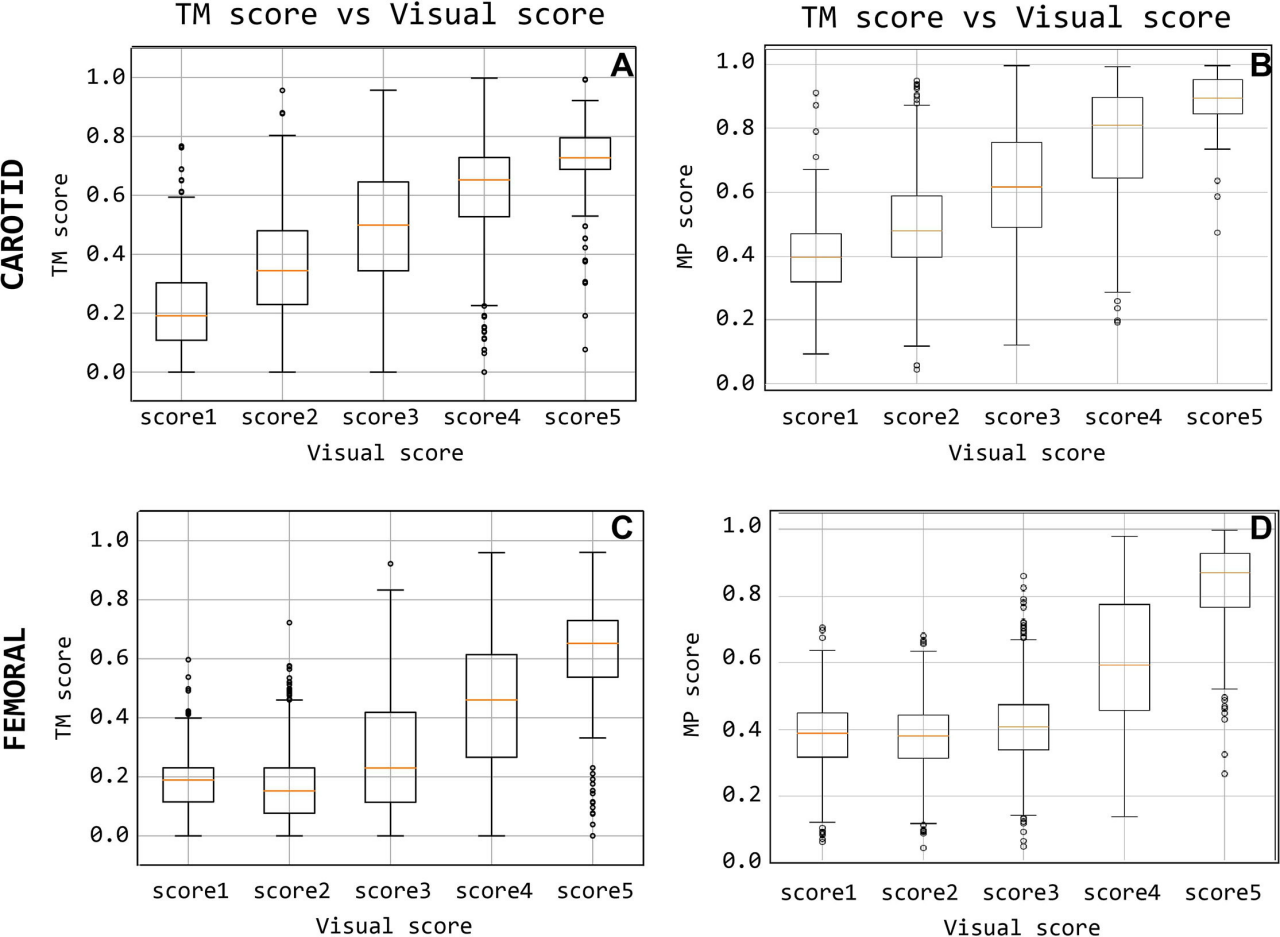
Quality score comparison between visual score and the template-matching-derived and matrix-profile-derived. Subfigures **(A,C)** display the score based on the template matching and **(B,D)** quality score based on the matrix profile.

The level of agreement obtained between *Q*_TM_ and *Q*_vis_ on the CC recordings dataset, with template matching adopting a 200 ms template length, is presented in Figure 10. This suggests that the median of the *Q*_TM_ values, computed on traces that have a *Q*_vis_ = 3, could be adopted as a threshold value for the automatic quality checking of an LDV trace (i.e., in the case under study, traces with a *Q*_TM_ > 0.5 could be considered of adequate quality; note that, manually setting these thresholds is not required for the logistic regression models since this is implicitly learned in the training).

**Figure 11:**
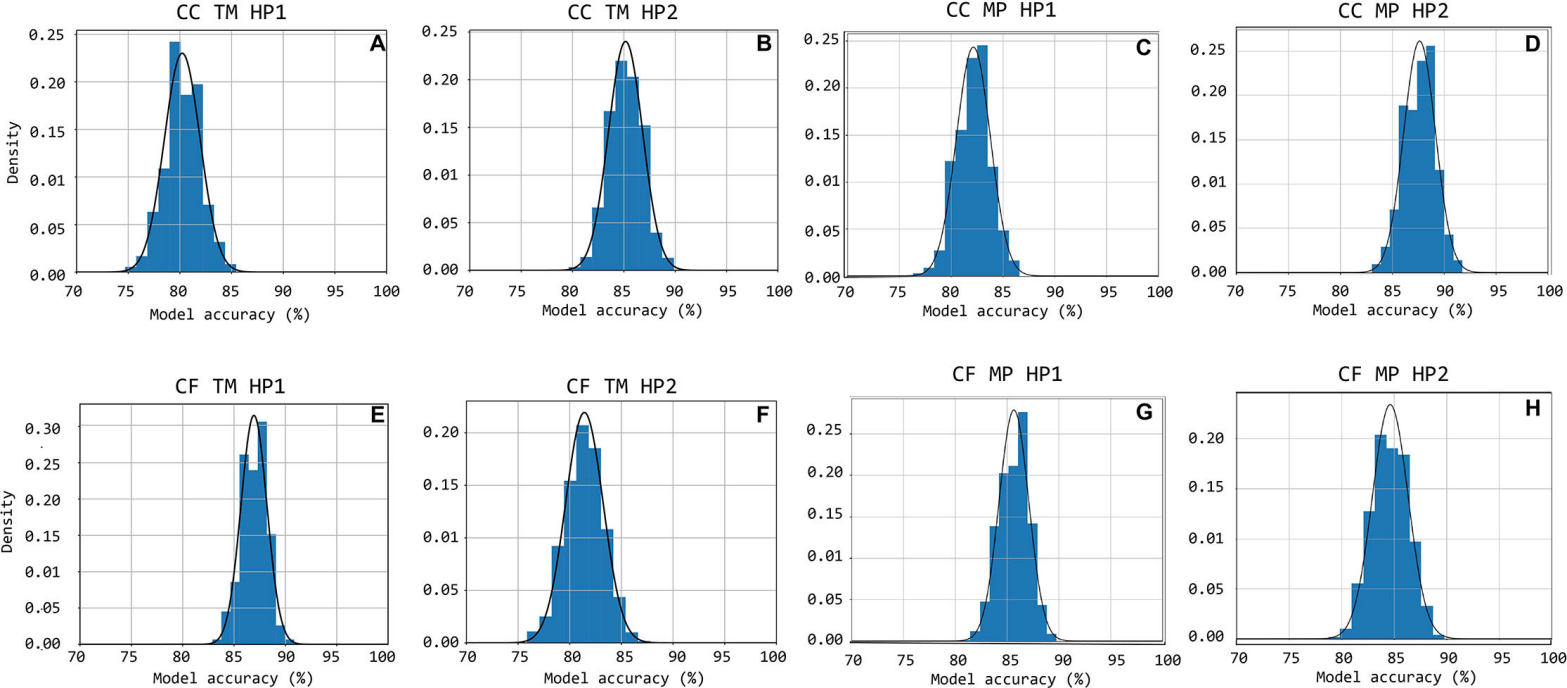
Accuracy distributions for 1,000 random train-test set partitions and subsequent logistic regression models trained. The accuracy distribution is shown cumulatively through bar-charts with the equivalent Gauss-curve plotted on top of it. Subfigures **(A–H)** show this for CC HP1, CC HP2, CF HP1, and CF HP2 cases, respectively.

The accuracy distributions of logistic regression models trained on quality scores derived from template-matching are displayed in Figure 11. On average, the accuracy on traces acquired using handpiece 2 is higher than handpiece 1 (85 ± 1.6% and 80 ± 1.70%, respectively; the results are summarized in **Table 4**).

#### 3.2.2. Carotid-Femoral Measurements

From the analysis carried out on the complete CF dataset, it emerged that using the carotid template of 500 ms length guarantees the best performance in terms of specificity, setting the cross-correlation threshold to 0.56 and the minimum number of detected heartbeats per trace to 10 (AUC = 0.89, sensitivity 77%, specificity 92%; template of 400 ms length: AUC = 0.88, sensitivity 76%, specificity of 91%; template of 300 ms length: AUC = 0.87, sensitivity 72%, specificity 92%). The confusion matrices are shown in Table 3 for each template.

**Table 3:** Confusion matrices of signal classification done by the hand-engineered classification model constructed with template matching.

	**Template of 300 ms**		**Template of 400 ms**		**Template of 500 ms**	
**Femoral recordings**	**TM score 0(%)**	**TM score 1(%)**	**TM score 0(%)**	**TM score 1(%)**	**TM score 0(%)**	**TM score 1(%)**
Score 1	96	4	96	4	96	4
Score 2	91	9	89	11	91	9
Score 3	68	32	64	36	65	35
Score 4	33	67	28	72	27	73
Score 5	8	92	6	94	7	93

*Signals classified in this table were measured at the femoral and the templates used were the femoral population templates*.

As for the carotid traces, the performance of the template matching algorithm was based on the specificity values, in order to remove the bad quality signals. More in detail, the template matching strategy shows excellent performance for the classification of visual scores 1 and 5 (accuracy of 96 and 93%, respectively), while the accuracy decreases for class 2 and class 4 (91 and 72%, respectively). In the femoral case, the method classified a majority of LDV traces with a visual score of 3 (borderline) as inadequate. Indeed, considering the template of 500 ms, the template matching method classified 65% of score 3 as inadequate signals and the other 35% (borderline)as adequate.

The level of agreement obtained between *Q*_TM_ and *Q*_vis_ on the CF recordings dataset, using the 500 ms template length, is shown in Figure 10C. The results indicate that from the median *Q*_TM_ values scored *Q*_vis_ = 3, a threshold value could be adopted for the automatic quality checking of the LDV trace (i.e., in the case under study, traces with a *Q*_TM_ > 0.23 could be considered of adequate quality; again, this threshold is not required when working with the logistic regression models.)

The accuracy distributions of logistic regression models trained on quality scores derived from template-matching are displayed in Figure 11. On average, the accuracy on traces acquired using handpiece 1 is higher than handpiece 2 (87 ± 1.3% and 81 ± 1.9%, respectively; the results are summarized in Table 4).

**Table 4:** The table contains the average performance of the logistic regression models trained on features derived by both template matching and matrix profile methods.

	**Template matching**		**Matrix profile**	
**Accuracy**	**Average(%)**	**Std(%)**	**Average(%)**	**Std(%)**
Carotid-carotid hp1	80	1.75	82	1.64
Carotid-carotid hp2	85	1.63	88	1.53
Femoral-carotid hp1	87	1.31	86	1.43
Femoral-carotid hp2	81	1.96	85	1.71

*Results are shown per handpiece of the measuring device. Average classification accuracy and its SD are given*.

### 3.3. Matrix Profile

On good quality data, i.e., those visually scored at 4 or 5, the matrix profile technique was able to include nearly all heartbeats in the motif. On poor quality data, the matrix profile was unable to identify most heartbeats because of noise or artifacts in the measurement. On some measurements that contain pure noise, the matrix profile picked up random noisy waveforms that were less prevalent and differed much compared to the desired foot-of-the-wave waveform.

#### 3.3.1. Quality Metric Results

The signals measured at the carotid measuring site were given a matrix profile-derived quality score that is compared with their visual scores in Figure 10B. A positive, linear relation between the two scoring methods is observed for the carotid-carotid database. The same information is shown for the femoral measuring site in Figure 10D. The difference between poor and good quality signals is apparent. Signals with visual scores 1, 2, or 3 have significantly lower *Q*_MP_ than those with visual scores 4 or 5.

#### 3.3.2. Logistic Regression Models Performance

Figures 10C,D,H show the accuracy distributions of the repeated logistic regression model-training experiment for signals measured in the neck with the different handpieces. All accuracy averages are above 80 with 82% (±1.64%) and 88% (±1.53%) for carotid-carotid recordings with handpieces 1 and 2, respectively. For carotid-femoral recordings, carotid data recorded with handpiece 2 yielded an accuracy of 85%± 1.71%. The distributions were assumed to be normally-distributed after a Shapiro-Wilk test and thus the Gauss-curves are drawn onto the subfigures of Figure 11.

The same data for the femoral data (measured with handpiece 1 during carotid-femoral recordings), is shown in Figure 11G. Average accuracy of 86% with a SD of 1.43% is observed. All accuracy statistics of the different measurement situations are summarized in Table 4.

### 3.4. Signal Quality vs. Physiological Variables

**Figure 12:**
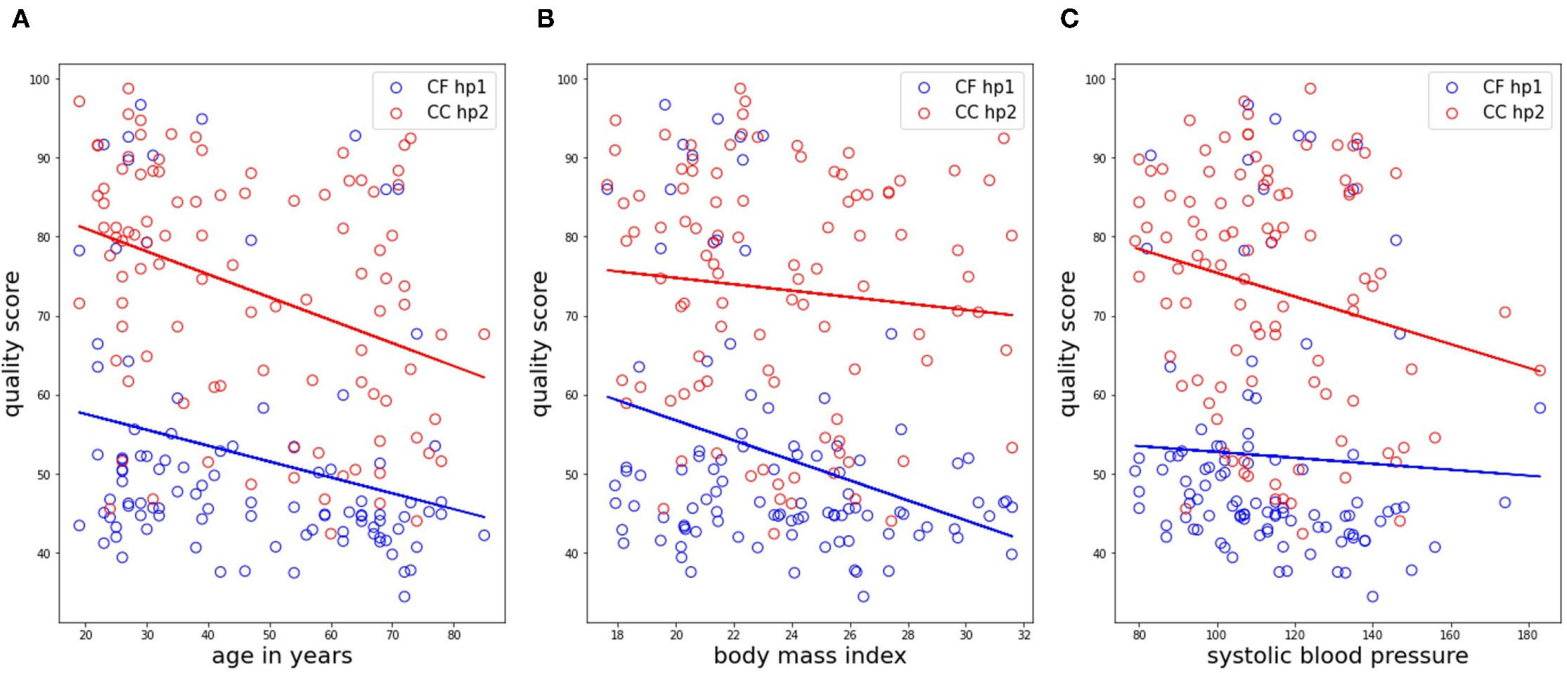
Correlation analyses of the matrix profile-based quality score with age, BMI and systolic blood pressure, shown in subfigures **(A–C)**, respectively. Only data in the CC HP2 and CF HP1 cases is shown. Trendlines of the data are also drawn.

The results from the correlation analysis between *Q*_*M*_*P* and age, BMI, and systolic blood pressure are shown in Figure 12 for the femoral data (carotid-femoral recording, handpiece 1; CF hp1) and the carotid-carotid recordings with handpiece 2 (CC hp2) showing the strongest trends. Significant negative correlations were found between age and *Q*_MP_ for CF hp1 (*r* = –0.253, *P* < 0.05) and CC hp2 (*r* = –0.365, *P* < 0.001). The correlation with BMI (Figure 11B) was significant only for the femoral recording (*r* = –0.304, *P* < 0.01) while the correlation with systolic blood pressure was significant only for CC hp2 (*r* = –0.206, *P* < 0.05)(Figure 11C). In a multivariate regression model including both age and systolic blood pressure, the correlation between carotid signal quality and systolic blood pressure was no longer significant (due to the correlation between age and systolic blood pressure). In contrast, in a multivariate model of femoral signal quality, both age and BMI remained significantly correlated with signal quality. The same relations are found when repeating the analysis with *Q*_vis_ or *Q*_MP_ (data not shown).

## 4. Discussion

The potential of LDV for non-contact measurement of physiological (cardiovascular) signals has been reported since about 2000 in explorative studies (Pinotti et al., 1998; Morbiducci et al., 2007a; Kaplan et al., 2012; Rohrbaugh et al., 2013) making use of bulky industry-time devices, and the technique has been suggested for measurement of carotid-femoral PWV by De Melis et al. (2008). An important technological breakthrough to enable LDV-based measurements in a clinical setting is the use of silicon photonics to miniaturize and integrate the optical components onto chips (Li et al., 2013) that are easily built-in into hand-held devices as the CARDIS prototype used in this study. That prototype was used in a clinical feasibility study where measurements were performed on the carotid and femoral artery, and we previously reported on the agreement of LDV-based carotid-femoral PWV with a reference method (Marais et al., 2019). In that article, data were processed off-line and algorithms for foot detection relied on the ECG and gating was applied on carotid and femoral tracings to ensure identification of the correct characteristic points on the waveforms. Further developments aim for ECG-independent measurements and will require a more stringent quality assessment in real-time application to ensure that data is captured from which transit times can be derived. Unlike the CARDIS device, future versions of the device will provide real-time feedback on signal quality and valid measurements will only be accepted after a minimal number of data samples have been retrieved from signals passing predefined quality criteria. In this study, we explored two possible strategies for such quality assessment, template matching and matrix profile, and benchmarked them using visual scoring as reference.

The visual grading was done by what we considered an expert observer but is inherently subjective. The graphical user interface that was developed showed all data within one single window for reasons of efficiency but inevitably leads to a weighed appreciation where data from different channels do get, to some extent, a degree of relative scoring. This mainly applies to the scores good (4)-excellent (5) where recordings of certain channels could have likely received different rankings if they had been individually assessed without the knowledge of the signal on the other channels. This remark may also pertain to the grade borderline. As future use of the device will target acquiring the best possible signals in a given subject, we particularly focused on signals graded 4 or 5. Figure 9 provides a visual overview of observed quality across the complete database. Each handpiece of the device is equipped with 6 channels in line, spanning 2.5 cm with the aim to have minimally one channel that detects a strong signal. It is clear that channel 1 on handpiece 1 systematically yields very low scores, which was attributed to a hardware problem with the inadequate alignment of the optical components during device assembly. For carotid measurements, the middle channels 3 and 4 yielded the highest quality signals (as expected), but this shifted to channels 2 and 3 for handpiece 2. Also, the overall signal quality was slightly higher for handpiece 2. We speculate that the use of the spacer underneath handpiece 1 may contribute to the difference in signal quality between both handpieces. These data can be compared to the data from handpiece 2 during carotid-femoral measurement, where handpiece 2 is now equipped with a spacer (refer to Figure 9 for the measuring configurations). The mean signal quality is now in the same range as it was for handpiece 1 on the local carotid measurements. An extra factor, however, is the fact that carotid-femoral measurements are technically more demanding, requiring the simultaneous acquisition of signals at 2 distinct locations. The same conclusions can be drawn on the basis of the automatically calculated sores *Q*_TM_ and *Q*_MP_.

In essence, one very good to excellent channel recording on each of the handpieces should guarantee a reliable transit time estimation from one handpiece to the other. This was achieved in 27% of the local carotid datasets and in 13% of the carotid-femoral datasets mainly due to the suboptimal femoral recording that is more challenging due to the fact that the operators have to manipulate two sensors on two distinct locations as well as the deeper positioning of the femoral artery leading to weaker signals. That does not imply that the remaining datasets cannot be processed (especially when the ECG is available; refer to Marais et al., 2019) or that LDV would not be suitable as a measuring technique; we just speculate that these results can be drastically improved with real-time feedback on the signal quality upon measurement.

The main objective of this study was to explore different methods for an automated signal quality assessment, where we first explored template matching. The template should minimally contain the foot fingerprint of the wave, apparent on both the carotid and femoral measuring locations. That pattern turned out to be fairly robust across the tested population. Even though the amplitude of acceleration signals was lower at the femoral measuring site, the pattern of the foot is quite similar on both measuring locations. A practical choice that has to be made is the length of the template. For carotid signals, it may be relevant to extend the template such that it also encompasses the dicrotic notch. We preferred the shorter template of 200 ms (which does not extend beyond the dicrotic notch) as the time delay between the wave's foot and the dicrotic notch is not constant but varies in between subjects and also within one subject from cycle to cycle due to physiological variations in blood pressure and heart rate. The shorter template was found to result in a somewhat higher specificity in correctly classifying poor signals, but overall, the performance of the carotid templates with different lengths was not very different, as can be observed from the confusion matrix (Table 2). On the other hand, for the femoral artery, we preferred the longest template of 500 ms which should detect epochs characterized by one prominent peak, the foot of the wave, followed by a long tail of low amplitude signals.

We then determined optimal thresholds levels for the magnitude of the cross-correlation and the number of detected beats using ROC analysis, whereby we maximized the classification performance of a binary classifier on the basis of *Q*_TM_. In this exploratory study, that analysis was done on the complete database and further optimizations should be done on the used features and repeating the analysis with separate training and testing data set. Using the resulting thresholds, the agreement between *Q*_TM_ and *Q*_vis_ was overall satisfactory. The logistic regression model analyses learned that a template matching approach is a valuable option to automatically classify signal quality as acceptable or not acceptable with an accuracy of over 80%.

As a second method, we considered the matrix profile as a technique to identify recurring patterns in the LDV-measurements in an automatic manner (Zimmerman et al., 2019), with very few control parameters. The potential advantage of a matrix profile approach over template matching is that no prior knowledge is required on the shape of the signal feature that one is looking for. Also, using the matrix profile allows the generation of a “user-dependent” template *in situ*. Signal quality was quantifiable using features of the motifs found by the matrix profile and combined into the quality metric *Q*_TM_, which showed a good agreement with the ground truth of visual scores as can be observed from Figure 10.

As for the template matching approach, the average accuracy of a logistic regression model trained and tested on features derived from motifs provided by the matrix profile technique is in all cases higher than 80%. Overall, only relatively small differences are observed between the two techniques. Both techniques perform similarly well which suggests that both, or a combination of the two, can be used for classifying new, future data into “bad, unusable” or “good, usable.” This allows us to state that a logistic regression model suffices, along with the signal features and techniques that are considered, to accurately assess incoming data in future real-time applications.

In our logistic model training, we purposely discarded datasets visually labeled “borderline” (score 3) as these data were simply hard to classify visually in an unequivocal way. That difficulty is relatively well reflected in the values of the quantitative metrics for these signals (Figure 10) and the performance of the classifiers as quantified by the confusion matrix (Tables 2, 3). Especially for the carotid artery, automated classification leads to a close to fifty-fifty percent labeling of data as acceptable or not acceptable. For the femoral recordings, there is a larger tendency to classify signals with a visual score of 3 as not acceptable. This is in line with our own perception that femoral data may have received higher scores than carotid data of similar quality and underlines the need for objective tools to score signal quality.

Interestingly, the quality score, exemplified by *Q*_MP_, correlates negatively with age and especially with BMI when signals are measured in the groin on the femoral artery. This observation supports the operators impression that measuring good quality LDV-signals on more obese subjects is consistently more challenging. The deeper the positioning of the artery and the more surrounding tissue, the stronger the signal attenuation. Such relation with BMI was absent for neck recordings. Also, skin inelasticity or thickness is expected to play a role in the transmission of intra-arterial vibrations and likely contributed to the observed negative correlation between and signal quality at the carotid and femoral locations in the study populations. The negative correlations between signal quality and age for carotid-carotid recordings with handpiece 2 were less strong, and were not found for the other carotid recordings (carotid-carotid handpiece 1 or carotid-femoral handpiece 2 recordings). A possible explanation may be the use of the spacer for these latter measurements, which may mechanically interfere with the transmission of the vibrations from within the artery to the skin and exert an effect on the recordings. Overall, this effect is considered minor, but it may nonetheless be a factor contributing to observed differences in the recordings.

The CARDIS prototype has a laser wavelength of 1,550 nm which is insufficiently reflected by the skin. We, therefore, attached retroreflective patches to the skin at the measurement locations to enhance reflection. The next-generation prototype aims for measurements without the retroreflective patch to facilitate practical use. A wavelength of 1,300 nm, for which there is a relative peak in skin reflectance (Rockwell and Goldman, 1974), will be used but the impact of skin pigmentation or sweating on data quality will have to be investigated.

In this study, signal quality was assessed off-line on 20 s recordings. Future developments will focus on real-time assessment of data quality as data is being captured and where the considered techniques will be used for epoch detection and subsequent quality quantification. Although a template-matching approach has the benefit that prior knowledge can be used to assess incoming data from the start, we assume that both techniques provide similarly useful features and that both are suitable for real-time implementation. It may be an option to hybridize the two techniques to come to a stronger, even more robust algorithm when implementing them into the device.

## 5. Conclusion

In conclusion, template matching and matrix profiling are methods suitable for the automated assessment of the signal quality of acceleration data measured from the skin in the neck and groin using laser Doppler velocimetry. Both methods allow to identify epochs in a data stream and provide quantifiable features that can be combined into a quality score or be used as input for logistic regression models for automated classification of signals as acceptable or not acceptable. Models based on both methods yielded an accuracy of minimally 80% in our CARDIS database of carotid and femoral recordings, reaching as high as 87% for the femoral data.

## Data Availability Statement

The original contributions presented in the study are included in the article/supplementary files, further inquiries can be directed to the corresponding author.

## Ethics Statement

The studies involving human participants were reviewed and approved by HEGP Ethics Committee. The patients/participants provided their written informed consent to participate in this study.

## Author Contributions

The manuscript was written by SS, SB, PS, and NM, as they performed the analysis that is documented. SS and SB contributed equally to the analysis and writing of the text. All authors are included as they provided invaluable work regarding the design of the device and conducting the measurements.

## Funding

The research was funded by the European Union's Horizon 2020 Research and Innovation Programme under grant agreement nos. 644798 (CARDIS) and 871547 (INSIDE).

## Conflict of Interest

The authors declare that the research was conducted in the absence of any commercial or financial relationships that could be construed as a potential conflict of interest.

## Publisher's Note

All claims expressed in this article are solely those of the authors and do not necessarily represent those of their affiliated organizations, or those of the publisher, the editors and the reviewers. Any product that may be evaluated in this article, or claim that may be made by its manufacturer, is not guaranteed or endorsed by the publisher.
